# Adjuvant effect of docetaxel on the immune responses to influenza A H1N1 vaccine in mice

**DOI:** 10.1186/1471-2172-13-36

**Published:** 2012-07-07

**Authors:** Jian Chen, Lin Yuan, Qing Fan, Fei Su, Yu Chen, Songhua Hu

**Affiliations:** 1 Department of Veterinary Medicine, College of Animal Sciences, Zhejiang University, Hangzhou, Zhejiang, 310058, China; 2 Institute of Microbiology, Academy of Jiangxi Province, Nanchang, Jiangxi, 330029, China; 3 Center of Experimental Animals, Zhejiang Academy of Chinese Materia Medica, Hangzhou, Zhejiang, 310007, China

**Keywords:** Docetaxel, Adjuvant, Influenza, H1N1, Th1/Th2

## Abstract

**Background:**

Vaccination remains one of the most effective approaches to prevent the spread of infectious diseases. Immune responses to vaccination can be enhanced by inclusion of adjuvant in a vaccine. Paclitaxel extracted from the bark of the Pacific yew tree *Taxus brevifola* was previously demonstrated to have adjuvant property. Compared to paclitaxel, docetaxel is another member of taxane family, and is more soluble in water and easier to manipulate in medication. To investigate the adjuvant effect of this compound, we measured the immune responses induced by co-administration of a split inactivated influenza H1N1 vaccine antigen with docetaxel.

**Results:**

When co-administered with docetaxel, lower dose antigen (equivalent to 10 ng HA) induced similar levels of IgG and IgG isotypes as well as HI titers to those induced by higher dose antigen (equivalent to 100 ng HA). Docetaxel promoted splenocyte responses to H1N1 antigen, ConA and LPS, mRNA expressions of cytokines (IFN-gamma, IL-12, IL-4 and IL-10) and T-bet/GATA-3 by splenocytes. The enhanced immunity was associated with up-expressed microRNAs (miR-155, miR-150 and miR-146a) in docetaxel-stimulated RAW264.7 cells. Docetaxel promoted similar IgE level to but alum promoted significantly higher IgE level than the control.

**Conclusion:**

Docetaxel has adjuvant effect on the influenza H1N1 vaccine by up-regulation of Th1/Th2 immune responses. Considering its unique vaccine adjuvant property as well as the safe record as an anti-neoplastic agent clinically used in humans during a long period, docetaxel should be further studied for its use in influenza vaccine production.

## Background

The current strategy for prevention of annual seasonal influenza is primarily based on the trivalent inactivated vaccine, which consists of split viral envelopes with the protein hemagglutinin (HA) as the main vaccine antigen [1,2]. Although the method has been utilized for many years, vaccination remains one of the most effective approaches, not only to prevent the spread of the influenza virus but also to mitigate the severity of illness and the impact of the disease [3] Since the rapid spread of the swine-origin influenza A (H1N1) 2009 pandemic worldwide, the rapid implementation of a vaccine has become a global priority.

A previous investigation found that vaccination with recent seasonal influenza vaccines provided little or no cross-reactive antibody protection against 2009 pandemic influenza A (H1N1) in any age groups [4]. The lack of cross-protective immunity between the pandemic and seasonal influenza virus strains highlighted the urgency of rapid vaccine development. The present global production capacity of trivalent seasonal influenza vaccine is about 876 million doses per year. With a world population of more than 6.5 billion people and the probability that two vaccine doses should be administered in a largely naive population, it should be predicted that about 13 billion doses of pandemic vaccine would be needed for adequate pandemic preparedness. The yield of virus in eggs or cell cultures is another important determinant for the amount of vaccine doses that can be manufactured. In spite of the WHO global pandemic influenza action plan to increase the potential supply of pandemic influenza vaccine [5], the production of sufficient pandemic vaccine to immunize the world’s population would significantly exceed the existing manufacturing capacities.

To effectively vaccinate the high-risk population against pandemic influenza, two challenges are to produce sufficient quantities of vaccine during a short period and to induce significant immunogenicity and cross-protective immunity after vaccine injections [6-8]. Luckily, both purposes can be achieved by using adjuvant to elicit a strong and broadened immune response. Although many materials have been reported having adjuvant property, alum (a term for aluminum-based mineral salts) is the first adjuvant approved by the U.S. Food and Drug Administration (FDA) in the influenza vaccines for human use. However, highly heterogeneous, difficult to manufacture in a consistent and reproducible manner, and a boost injection required to generate protection limited alum in influenza vaccine use [9,10]. It is also found that certain antigens do not adsorb well onto alum due to the presence of the same charge on the adjuvant and antigens [11]. MF59 is an oil-in-water emulsion adjuvant, which has been formulated in vaccines licensed for human use [12]. Although adverse effects of MF59 are rare, the events such fever, chills, malaise, myalgia, arthralgia, nausea, headache and rash have been found after immunization of MF59-adjuvanted vaccines [13,14]. Therefore, searching for new vaccine adjuvants remain an interesting topic.

Paclitaxel, a member in the taxane family, was initially extracted from the bark of the Pacific yew tree *Taxus brevifola* in early 1960s and its structure was confirmed in 1971 [15]. At the late 1970s, paclitaxel was discovered able to blocks mitosis and cause the death of cancer cells by binding to and stabilizing microtubules [16,17]. In 1992, the drug was approved for the treatment of advanced ovarian cancer, and then has been successfully used in other solid tumors [18,19]. The drug has a safe record in humans for almost 20 years. Based on the TLR4 agonist activity of paclitaxel at a low dose for stimulation of proinflammatory mediator release from isolated macrophages, it was previously demonstrated that paclitaxel has an adjuvant effect on the immune responses [20,21]. When co-administrated with paclitaxel, OVA induced significantly higher IgG, IgG subclass and IgM responses in association with upregulation of mRNA expression of T-bet/GATA-3 than when OVA was immunized alone [21].

Docetaxel is another member of the taxane family. Compared to paclitaxel, docetaxel is more soluble in water, and easier to manipulate in medication. Docetaxel has also been found to have immunomodulatory properties. Garnett et al. recently reported that intraperitoneal injection of docetaxel after subcutaneous inoculation of a recombinant poxviral vaccine significantly enhanced the immune response in a mouse model [22]. Present study was designed to investigate if co-administration of a split inactivated influenza H1N1 vaccine antigen with docetaxel could enhance the immune responses by measuring serum specific antibody responses, total IgE, hemagglutination inhibition titers (HI), lymphocyte proliferation as well as mRNA of cytokines and transcription factors produced by splenocytes in Balb/c mice. Dose-sparing effect of the influenza antigen was also evaluated when docetaxel was administered with the antigen.

## Results

### Serum vaccine-specific IgG and IgG isotypes

**Figure 1:**
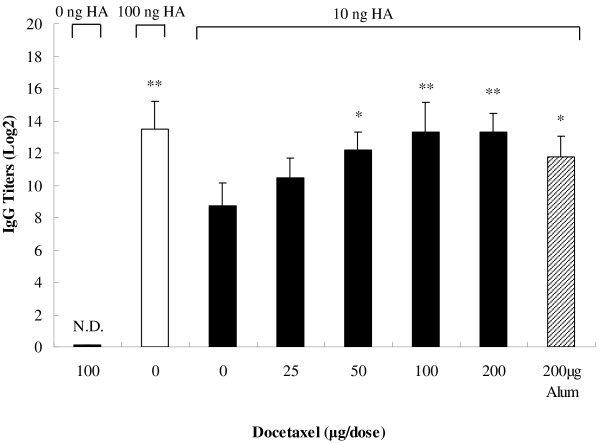
**Serum IgG titers elicited by inactivated H1N1 influenza virus antigen.** Mice (8 animals/group) were subcutaneously injected at weeks 0 and 3 with 100 μg docetaxel, inactivated H1N1 influenza virus vaccine (equivalent to 10 or 100 ng HA) or inactivated H1N1 influenza virus vaccine (equivalent to 10 ng HA) adjuvanted with docetaxel (25, 50, 100 or 200 μg) or alum (200 μg). Blood samples were collected 2 weeks after the second immunization for analysis of IgG titers by indirect ELISA. Values above the cut-off background level, mean value of sera from saline-immunized mice (negative control) multiplied by a factor of 2.1 were considered positive. Values represent mean ± S.D. Titers were depicted as reciprocal end-dilutions. Significant differences with 10 ng HA groups were designated as *P value < 0.05 and **P < 0.01. N.D. indicates that IgG titer was not detectable at the lowest dilution of serum.

Serum specific IgG and the IgG subclasses were measured by an indirect ELISA to evaluate the adjuvant effect of docetaxel on the humoral immune responses. Figure 1 shows that vaccine containing 10 ng HA (referred as 10 ng HA hereafter) induced significantly lower vaccine-specific IgG titers than 100 ng HA (*P* < 0.05). However, the IgG titer induced by 10 ng HA in combination with docetaxel (100 or 200 μg) was 23 times higher than that induced by the same amount of HA (*P* < 0.01) and similar to the IgG titer elicited by 100 ng of HA (*P* > 0.05). IgG titers were dose-dependent on the amount of docetaxel, and reached the highest when docetaxel was at 100 and 200 μg but was not significantly increased when docetaxel increased from 100 μg to 200 μg. Although IgG titer in alum-adjuvanted group was significantly higher than no adjuvanted group, it was significantly lower than that in the group adjuvanted with 100 or 200 μg of docetaxel. As no OD values of the sera from docetaxel-injected mice were recorded above 2.1 × mean value of the sera from saline-injected mice (negative controls), IgG titer was actually undetectable in docetaxel-injected group.

**Figure 2:**
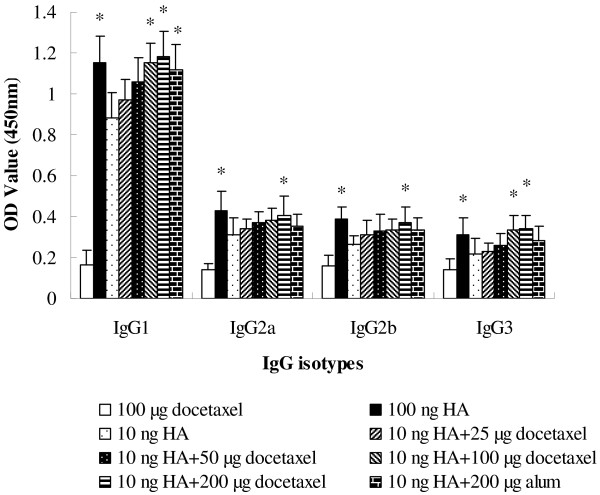
**Serum specific IgG1, IgG2a, IgG2b and IgG3 levels.** Mice (8 animals/group) were subcutaneously injected at weeks 0 and 3 with 100 μg docetaxel, inactivated H1N1 influenza virus vaccine (equivalent to 10 or 100 ng HA) or inactivated H1N1 influenza virus vaccine (equivalent to 10 ng HA) adjuvanted with docetaxel (25, 50, 100 or 200 μg) or alum (200 μg). Blood samples were collected 2 weeks after the second immunization for IgG isotype analysis by indirect ELISA. Values represent mean ± S.D. Significant differences with 10 ng HA groups were designated as *P < 0.05 and **P < 0.01.

Figure 2 indicates that 100 ng of HA induced higher vaccine-specific IgG1, IgG2a, IgG2b and IgG3b responses than 10 ng of HA (*P* < 0.05). Supplement of docetaxel (100 or 200 μg) but not alum in 10 ng of HA significantly enhanced the isotypes IgG1, IgG2a, IgG2b and IgG3b responses when compared with the group injected with same amount of HA without adjuvant.

### HI titers

**Figure 3:**
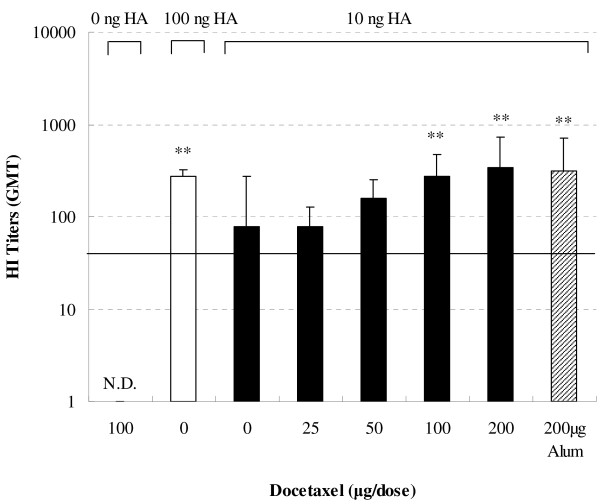
**Serum HI titers.** Mice (8 animals/group) were subcutaneously injected at weeks 0 and 3 with 100 μg docetaxel, inactivated H1N1 influenza virus antigen (equivalent to 10 or 100 ng HA) or inactivated H1N1 influenza virus antigen (equivalent to 10 ng HA) adjuvanted with docetaxel (25, 50, 100 or 200 μg). Blood samples were collected 2 weeks after the second immunization for hemagglutination inhibition test. Values represent geometrical mean titer (GMT) ± S.D. Significant differences with 10 ng HA group was designated as *P < 0.05 and **P < 0.01. N.D. indicates that HI titer was not detectable at the lowest dilution of serum. The solid horizontal line represents an HI titer of 40.

To investigate the effect of docetaxel on serum HI response, mice were immunized twice s. c. and serum HI titers were determined. Figure 3 indicates that 10 ng of HA induced lower HA-specific HI titer than 100 ng of HA vaccine (*P* < 0.05). Co-administration of virus antigen (equivalent to 10 ng HA) with docetaxel at the dose more than 50 μg induced higher HI titers than the same amount of virus antigen without adjuvant. HI titer induced by 10 ng HA antigen adjuvanted with 100 or 200 μg of docetaxel was similar to that induced by 100 ng of HA vaccine alone or 10 ng of HA vaccine adjuvanted with alum.

### Lymphocyte proliferative responses

**Figure 4:**
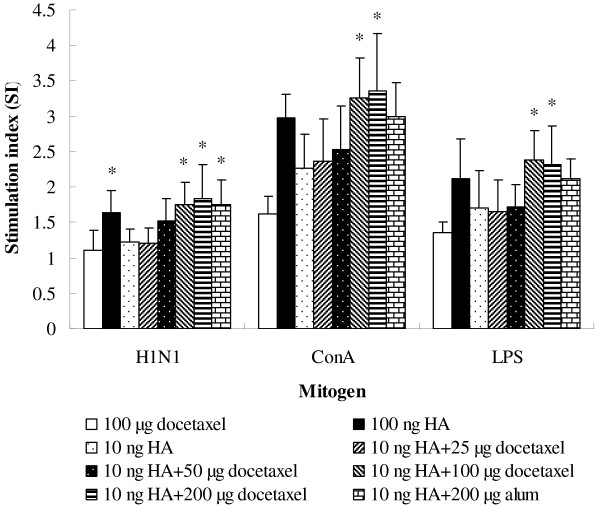
**Mitogen-stimulated proliferations of splenocytes.** Splenocytes isolated from mice (8 animals/group) received twice subcutaneous injections at weeks 0 and 3 of split H1N1 influenza virus antigen (equivalent to 10 and 100 ng HA) or inactivated H1N1 influenza virus antigen (equivalent to 10 ng HA) co-administrated with docetaxel (25, 50, 100 and 200 μg) or alum (200 μg) and docetaxel administrated alone. Splenocytes were prepared 2 weeks after the last immunization and cultured with ConA, LPS or RPMI 1640. Splenocyte proliferation was measured by the MTT method as described in the text, and is shown as a stimulation index (SI). Values represent mean ± S.D. Significant differences with 10 ng HA groups were designated as *P < 0.05 and **P < 0.01.

Figure 4 shows the effect of docetaxel on splenocyte proliferative responses to virus antigen, Con A and LPS stimulation. Higher splenocyte responses to vaccine (*P* < 0.05), Con A (*P* > 0.05) and LPS (*P* > 0.05) were found in the mice immunized with 100 ng HA than the mice immunized with vaccine containing 10 ng HA only. Supplement of docetaxel and alum in 10 ng HA vaccine enhanced splenocyte proliferative responses to the mitogens and vaccine. Significantly higher splenocyte responses to vaccine, ConA and LPS were detected in groups adjuvanted with docetaxel (100 and 200 μg) than in the control (immunized with vaccine containing 10 ng HA). Splenocyte proliferative responses to mitogen and vaccine stimulation were numerically higher in mice injected with 10 ng HA plus 100 or 200 μg of docetaxel than in mice injected with 100 ng HA alone. Proliferative responses to mitogens and HA of the splenocytes from mice injected with docetaxel only were at the same level as those from the mice injected with saline.

### Serum total IgE

**Figure 5:**
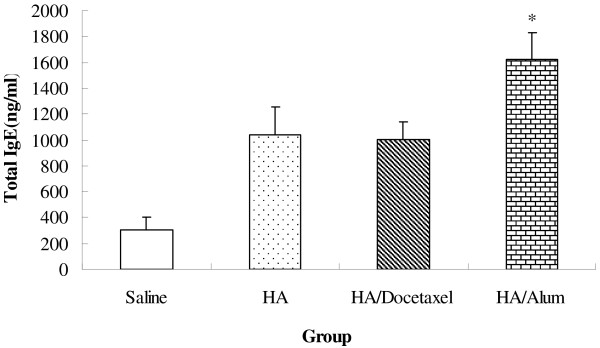
**Serum total IgE level in immunized mice.** Mice (8 animals/group) received twice subcutaneous injections at weeks 0 and 3 of saline, split H1N1 influenza virus antigen (containing 10 ng HA), or 10 ng HA plus 100 μg docetaxel or plus 200 μg alum. The mice were bled 2 weeks after the second immunization for analysis of total IgE. Significant differences with 10 ng HA groups were designated as *P < 0.05 and **P < 0.01.

Figure 5 shows that serum total IgE level in docetaxel-vaccine group was significantly lower than in alum-vaccine group (*P <* 0.05), but similar to the control (*P >* 0.05).

### Cytokines mRNA expression

**Figure 6:**
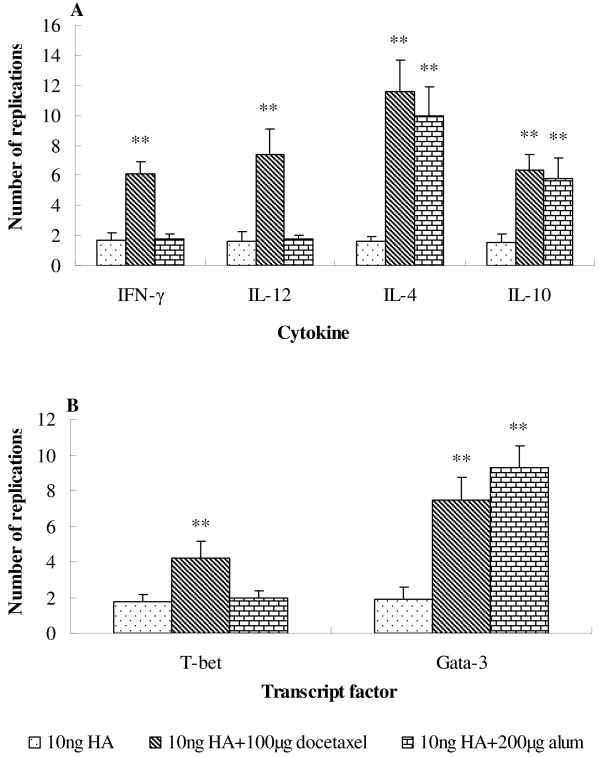
**Expression of cytokines and transcript factors mRNA by splenocytes.** Mice (8 animals/group) received twice subcutaneous injections at week 0 and 3 of inactivated H1N1 influenza virus antigen (equivalent to 10 ng HA) alone or with docetaxel (100 μg) or alum (200 μg). Mice injected with saline only were used as a calibrator group. Splenocytes were prepared 2 weeks after the second immunization and cultured with HA (1.32 μg/ml) for 10 h. mRNA expression of cytokines IFN-γ, IL-12, IL-4 and IL-10 **(A)** and transcript factors T-bet and Gata-3 **(B)** was analyzed by real-time PCR. Results are reported as the n-fold difference relative to cytokine mRNA expression of calibrator sample. Significant differences with 10 ng HA groups were designated as *P < 0.05 and **P < 0.01.

To investigate the effect of docetaxel and alum on cytokines mRNA expression, RNA was isolated from mouse splenocytes 2 weeks after inoculation and cytokines mRNA was quantified by real-time PCR (Figure 6A). When adjuvant was added, a remarkable change in the cytokine profile was observed. Alum (200 μg) and docetaxel (100 μg) induced different changes in the cytokines. Docetaxel + 10 ng HA induced higher mRNA expression of all cytokines than the control (10 ng HA alone) (*P* < 0.05), while alum + 10 ng HA triggered significantly higher IL-4 and IL-10 but not IFN-γ and IL-12 mRNA expression than the control (10 ng HA alone) (*P* < 0.05).

Docetaxel and alum induced different patterns of T-bet and GATA-3 mRNA expression as shown in (Figure 6B). Docetaxel + 10 ng HA induced significantly higher both T-bet and GATA-3 mRNA expression than the control without adjuvant (*P* < 0.05); alum + 10 ng HA induced significantly higher GATA-3 (*P* < 0.05) than the docetaxel group but similar T-bet mRNA expression to the control (*P* > 0.05 alone).

### MicroRNAs expressed by macrophages stimulated in vitro by docetaxel

**Figure 7:**
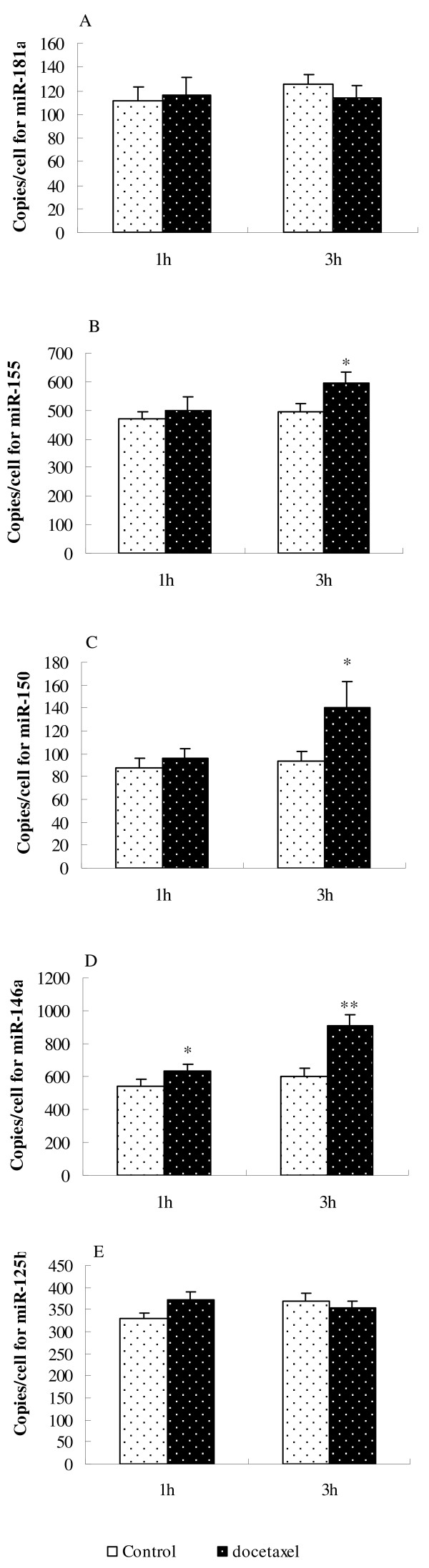
**MicroRNAs in murine macrophages (RAW264.7 cells).** Macrophages were cultured with saline (empty bars) or 0.8 μg/ml of docetaxel (dark bars) for the indicated lengths of time. Real-time PCR was performed to determination of miR-181a **(A)**, miR-155 **(B)**, miR-150 **(C)**, miR-146a **(D)** and miR-125b **(E)** as described in methods. Data are presented as copies ± SD/cell (n = 3). Significant differences with the control were designated as *P < 0.05 and **P < 0.01.

When murine macrophages were incubated with docetaxel at 0.8 μg/ml for 1 h, only miR-146a expression was significantly increased in comparison with the control (Figure 7D); 3 hours later, the expression of miR-155, miR-150 and miR-146a was significantly increased (Figure 7B, C, D) while miR-181a and miR-125b had no significant changes (Figure 7A, E).

## Discussion

Adjuvant properties of docetaxel have been demonstrated for inactivated H1N1 influenza vaccine in a mouse model in the present study. Co-administration of docetaxel with inactivated influenza virus H1N1 induced significantly higher serum specific IgG and the isotype responses, HI titer, splenocyte proliferation in response to ConA, LPS and HA than when influenza vaccine was immunized alone. In addition, significantly increased mRNA expressions of IFN-γ, IL-12, IL-4 and IL-10 by splenocytes in association with up-regulation of mRNA expression of T-bet/GATA-3 were observed in docetaxel-adjuvanted groups. Serum total IgE level in the docetaxel adjuvanted group was significantly lower than the alum-adjuvanted group. MiR-155, miR-150 and miR-146a are up-regulated in Raw 264.7 cells in response to docetaxel.

The mouse model has been used to study the immunity of a host against influenza infection for long time. For example, Cox et al. immunized mice with a split influenza virus vaccine, and observed that the inhibition of viral replication by immunization correlates high influenza specific serum IgG concentrations [23]. Caillet et al. immunized Balb/c mice with a H1N1 influenza vaccine using oil-in-water emulsion AF03 as an adjuvant, and found that the mice receiving AF03-adjuvanted vaccine had antigen-specific antibody titers 3- to 10-fold higher than that in animals administered antigen alone [24]. The antibody response elicited by antigen is dose-dependent. Caillet et al. reported that 0.3 μg influenza vaccine antigen induced lower HI tiers than 3 μg antigen in mice [24]. Similarly, we found that 10 ng HA antigen elicited significantly lower serum IgG and HI titers or the IgG isotypes than 100 ng HA antigen. However, supplement of docetaxel (50 to 200 μg) in 10 ng HA antigen significantly amplified IgG and HI titers, which were similar to the titers elicited by 100 ng HA when docetaxel was added at 100 or 200 μg as indicated in Figures 1 and 3, suggesting that the same level of the immune responses could be induced by smaller dose of antigen if docetaxel is used as an adjuvant in the production of vaccines. Garnett et al. significantly enhanced the immune response to a recombinant poxviral vaccine by injection of docetaxel at 500 μg [22]. In this study, antibody titers had no long significant changes when docetaxel was increased from 100 to 200 μg per dose.

IgG is the most plentiful immunoglobulin in the serum, and provides the considerable protection against most blood infectious agents. During a T-cell dependent immune response, a progressive change takes place in the principal immunoglobulin class of the specific antibodies. This subclass switch is influenced by T-cells and their cytokines. Data in Figure 2 indicated that docetaxel significantly increased the production of all IgG isotypes, which may be associated with simultaneously up-regulated gene expression of T-bet and GATA-3 (Figure 6B), leading to increased production of IFN-γ, IL-12, IL-4 and IL-10 by splenocytes (Figure 6A). While alum enhanced only IgG1 (*P* < 0.05) but not IgG2a, IgG2b and IgG3 (*P* > 0.05), which may be related to up-regulated gene expression of GATA-3, resulting in enhanced production of Th2 type cytokines such as IL-4 and IL-10 as shown in (Figure 6A). All these suggest that docetaxel activated both Th1 and Th2 while alum only triggered Th2 type immune responses.

Hemagglutinin has the capacity of binding to erythrocytes resulting in agglutination, which can be visually detected and thus used as assay read-out. The binding of HA to erythrocytes is inhibited by the addition of serum containing anti-HA antibodies. Thus, the concentration of anti-HA antibodies can be defined as HI titer by incubating serial dilutions of sera with HA antigen or whole virus [25]. In humans, HI titer of 1:40 or higher is normally considered protective [26]. Figure 3 showed that docetaxel increased HI titers, indicating that the protection capacity against influenza infection was increased in the immunized animals.

The lymphocyte proliferative response depends on the mitogen used. ConA stimulates T-cell whereas LPS stimulates B cell proliferation. Increased lymphocyte proliferation responses to ConA and LPS were found in docetaxel- and alum-adjuvanted groups (Figure 4), indicating that both T and B cells were activated. In order to induce antibody production, antigen-specific B lymphocytes should be triggered for clonal expansion. Significantly enhanced lymphocyte responses to H1N1 HA antigen, paralleled the increased HA-specific IgG responses in mice immunized with docetaxel- or alum-adjuvanted H1N1 vaccine.

Unlike paclitaxel, docetaxel does not bind to TLR4 nor stimulate proinflammatory cytokine responses [27]. Garnett et al. recently reported that docetaxel modulated CD4^+^, CD8^+^, CD19^+^, natural killer cells, and Treg populations and enhanced CD8^+^ functions [22]. The adjuvant activity of docetaxel may be related to its immunomodulatory effects. MicroRNAs (miRs) are a broad class of small non-coding RNAs (18–25 nucleotides) with crucial roles in regulation of gene expression. Previous studies have shown that miR-155, miR-150, miR-146a, miR-181a and miR-125b are involved in the innate immune reactions. Stimulation of monocytes with lipopolysaccharide (LPS) induced the expression of miR-146 and miR-155 [28]. MiR-155 and miR-125b were found to be up-regulated and down-regulated, respectively, in Raw 264.7 macrophages in response to LPS. The miR-150 has a dynamic expression profile during lymphocyte development, being highly expressed in mature B cells and T cells but not in their progenitors, its expression is then extinguished after further differentiation of naive T cells into the Th1 and Th2 subsets [29]. MiR-181a has been ascribed functions in hematopoietic differentiation and in T cell differentiation [30,31]. In this study, miR-181a, miR-155, miR-150, miR-146a and miR-125b were analyzed to identify microRNAs possibly involved in responses to docetaxel stimulation (Figure 7). Only miR-146a showed significant increase 1 hour after stimulation of RAW264.7 cells with docetaxel (Figure 7D). Three hours later, miR-155, miR-150 and miR-146a expressions were enhanced (Figure 7B, C, D), while miR-181a and miR-125b showed no significant change (Figure 7A, E). Increased expression of miR-155 and miR-146a has also been found in our previous study when RAW264.7 cells were stimulated with paclitaxel [21]. These suggested that stimulation manner of docetaxel may be different from that of LPS.

Safety should be taken into account when seeking adjuvant candidates. However, the safety of many drugs largely depends on how the drugs are used. Many drugs are safe at a small dose while becoming toxic when they are administered frequently at a higher dose. Compared to other potential vaccine adjuvant candidates reported in literatures, the toxicity of docetaxel is transparent as it has been clinically used for almost 20 years. When docetaxel was used as an antineoplastic agent, the side-effects such as short-lasting neutropenia and hypersensitive reactions were reported [32]. The other toxicities were hematopoietic (rats, mice, dogs and monkeys), gastrointestinal (dogs, monkeys) and neuromotor (mice), and are either usually mild in severity or easily treated or prevented [33]. In our study, the suggested dose of docetaxel for adjuvant purpose was 100 μg/mice, which was significantly lower than that recommended for cancer treatment (47 mg/kg).

The adjuvant effect of aluminum salts on influenza vaccine has been proven previously [34]. However, frequent use of alum-adjuvanted vaccines could be one of the reasons for IgE-mediated allergy due to activated Th2 immune response [35,36]. In the present study, alum but not docetaxel promoted the production of IgE significantly higher than that of mice immunized with HA only (Figure 5). The increased serum IgE level may be attributed to higher Th2 (IL-4, IL-10, GATA-3) and lower Th1 (IFN-γ, IL-12, T-bet) responses in mice injected with alum-adjuvated vaccine as indicated in Figure 6.

## Conclusions

In summary, docetaxel has an adjuvant effect on a split influenza A H1N1 vaccine by up-regulating Th1 and Th2 immune responses in a mouse model. When co-administered with docetaxel, 10 ng of H1N1 virus antigen (HA) induced similar level of IgG and IgG isotype responses as well as HI titers to those induced by 100 ng of HA. Docetaxel promoted splenocyte proliferative response to H1N1 antigen, ConA and LPS, mRNA expressions of cytokines (IL-4, IL-10, IL-12 and IFN-γ) and T-bet/GATA-3 by splenocytes. The enhanced immune responses may be associated with up-expressed microRNAs (miR-155, miR-150 and miR-146a) as detected in docetaxel-stimulated RAW264.7 cells. Docetaxel promoted similar IgE level to but alum promoted significantly higher IgE level than the control. Considering its unique vaccine adjuvant property as demonstrated here as well as the safe record clinically used in humans during a long period, docetaxel should be further evaluated for its use in vaccines.

## Methods

### Animals

Female Balb/c mice were purchased from Shanghai Laboratory Animal Center (SLAC) Co., Ltd. (Shanghai, China), and housed in polypropylene cages with sawdust bedding in hygienically controlled environment. The temperature was controlled at 24 ± 1°C and humidity at 50 ± 10%. Feed and water were supplied ad libitum.

### Ethic statement

All procedures related to the animals and their care conformed to the internationally accepted principles as found in the Guidelines for Keeping Experimental Animals issued by the government of China. One of the authors (Yu Chen) received license (No. X1003003) for management of experimental animals from the Office in Charge of Experimental Animals of Zhejiang Province. The Department of Veterinary Medicine has the relevant approval to carry out animal study in general and need not additional approval for this study.

### Antigen and adjuvant

Split inactivated influenza virus NYMCX-179A (H1N1) was kindly supplied by Zhejiang Provincial Center for Diseases Control and Prevention, which contained 132 μg/ml of hemagglutinin (HA) determined by a quantitative single-radial-immunodiffusion assay essentially as described by Wood et al. [37]. Docetaxel was purchased from Xi’an Hao-xuan Biotechnology Co., Ltd (Xi’an, China). Docetaxel was white powder with purity of 99.5%. Docetaxel was dissolved in absolute ethanol, polysorbate 80, saline (1:1:18) and sterilized by passing through a 0.22 μm filter. The endotoxin level in the solutions was less than 0.5 endotoxin unit (EU)/ml by a gel-clot Limulus amebocyte lysate assay (Bath no., Zhanjiang A & C Biological Ltd., Zhanjiang, China). Aluminum hydroxide Gel (Sigma, A8222) was used as the positive control adjuvant.

### Immunization

Seventy-two Balb/c mice (6 weeks of age) were randomly distributed into nine groups with 8 mice each. All animals were subcutaneously (s.c.) immunized twice with saline, docetaxel (100 μg), Ag (100 or 10 ng HA) or Ag (10 ng HA) + docetaxel (25, 50, 100 or 200 μg) at 3 week intervals, and the alum adjuvanted group as the positive control. Two weeks after the boost, blood samples were collected for measurement of serum HI titers using chicken red blood cells, Ag-specific IgG titers as well as IgG isotypes, and total IgE levels. Splenocytes were prepared for determination of cellular proliferation and production of IFN-γ, IL-12, IL-4, IL-10, T-bet and GATA-3. All the injection solution was in the volume of 0.1 ml per mouse.

### Measurement of Vaccine-specific IgG, IgG isotypes and total IgE

Serum samples were analyzed for measurement of vaccine-specific IgG titer and IgG isotype responses by indirect enzyme-linked immunosorbent assay as previously described by Song et al. [38]. Total IgE was measured using a mouse IgE ELISA quantitation kit (Biolegend, Cat. No. 432404) following the manufacturer’s instructions. The sensitivity of the assay was 0.1 ng/ml for mouse IgE. Serum was diluted 1:50 with 1× assay diluent.

### Hemagglutination inhibition assay

Serum HI titers were determined according to the protocol adapted from the CDC laboratory-based influenza surveillance manual [39].

### Lymphocyte proliferation assay

Spleen collected from the immunized Balb/c mice under aseptic conditions, in Hank’s balanced salt solution (HBSS, Sigma), was minced and passed through a fine steel mesh to obtain a homogeneous cell suspension. After centrifugation (380 × g at 4°C for 10 min), the pelleted cells were washed three times in PBS and resuspended in complete medium (RPMI 1640 supplemented with 0.05 mM 2-mercaptoethanol, 100 UI/ml penicillin, 100 μg/ml streptomycin and 10% heat inactivated FCS). Cell numbers were counted with a haemocytometer by trypan blue dye exclusion technique. Cell viability exceeded 95%. Splenocyte proliferation was assayed as described previously with some modification [40]. Briefly, splenocytes were seeded into a 96-well flat-bottom microtiter plate (Nunc) at 5.0 × 10^6^ cell/ml in 100 μl complete medium, thereafter concanavalin A (Con A, final concentration 5 μg/ml), LPS (final concentration 7.5 μg/ml), H1N1(final concentration 1.32 μg/ml) or medium were added giving a final volume of 200 μl. The plates were incubated at 37°C in a humid atmosphere with 5% CO_2_ for 2 days or 4 days. All the tests were carried out in triplicate. The cell proliferation was evaluated using MTT methods. Briefly, 50 μl of MTT solution (2 mg/ml) were added to each well 4 h before the end of incubation. The plates were centrifuged (1400 × g, 5 min) and the untransformed MTT was removed carefully by pipetting. To each well 150 μl of a DMSO working solution (192 μl DMSO with 8 μl 1 N HCl) was added, and the absorbance was evaluated in an ELISA reader at 570 nm with a 630 nm reference after 15 min. The stimulation index (SI) was calculated based on the following formula:

(1) SI=the absorbance value for mitogen cultures divided by the absorbance value for non−stimulated cultures.

### Quantification of target genes by real-time PCR

Splenocytes from the H1N1-immunized Balb/c mice prepared as described before were seeded into a 24-well flat-bottom microtiter plate (Nunc) at 5 × 10^6^ in 2 ml complete medium, thereafter 20 μl split virus antigen (equivalent to 2.64 ng HA) was added. The plates were incubated at 37°C in a humid atmosphere with 5% CO_2_. After 10 h treatment, cells were harvested by centrifugation (380 × g at 4°C for 10 min), and washed with ice-cold PBS, then subjected to RNA extraction. Splenocytes (1 × 10^7^) were lysed in 1 ml of RNAiso^TM^ Plus (Takara, China) reagent and the total RNA was isolated according to the manufacture’s protocol. The concentration of total RNA was quantified by determining the optical density at 260 nm. The total RNA was used and reverse transcription was performed by mixing 1 μg of RNA with 5 μl iScript reagent (Bio-Rad) in a DEPC-treated tube, thereafter nuclease-free water was added to a final volume of 20 μl. The reaction condition for reverse transcription was performed according to the manufacture’s protocol (5 min at 25°C, 30 min at 42°C, 5 min at 85°C, hold at 4°C).

Relative quantitation of GATA-3, T-bet and cytokines cDNA to β-actin message was conducted on ABI 7300 (PE Applied Biosystems, USA). The purpose of the house keeping gene (β-actin) is to normalize the PCRs for the RNA added to the reverse transcription reactions and to correct for differences in RT reaction efficiencies [41]. The PCR was performed in a dual PCR with primers and Tag-Man probes for the target gene and β-actin cDNA in the same reaction vessel. The primers and probes for target gene and β-actin (Table 1) were designed using Primer Express 3.0 (PE Applied Biosystems) according to the manufacturer’s directions and each primer was located in different exons of target genes. Probes for GATA-3, T-bet and cytokines were detected via a 5′ labeled with reporter dye (FAM) (Sangon Co., Ltd., Shanghai, China) and 3′ labeled with quench dye (BHQ-1) (Sangon Co., Ltd., Shanghai, China), while probes for β-actin were 5′ labeled with reporter dye (HEX) (Sangon Co., Ltd., Shanghai, China). The PCR products were identified by DNA sequencing.

**Table 1:** Sequences of primer and probe for quantitative RT-PCR of cytokine and transcription factor

**Gene**	**Primer sequence**
ß-Actin	Forward: 5′-AGCGGTTCCGATGCCCT-3′
	Reverse: 5′-AGAGGTCTTTACGGATGTCAACG-3′
	Probe:5′HEX-TCCTTCTTGGGTATGGAATCCTGTGGC-BHQ-13′
IL-4	Forward: 5′-GAGACTCTTTCGGGCTTTTCG-3′
	Reverse: 5′-CAGGAAGTCTTTCAGTGATGTGG-3′
	Probe: 5′FAM-CCTGGATTCATCGATAAGCTGCACC-BHQ-1 3′
IL-10	Forward: 5′-CCAGTTTTACCTGGTAGAAGTGATG-3′
	Reverse: 5′-CTTGCTCTTATTTTCACAGGGGAG-3′
	Probe: 5′FAM-CAGGCAGAGAAGCATGGCCCAGAAA-BHQ-1 3′
IL-12p40	IL-12p40 Forward: 5′-TTGCTGGTGTCTCCACTCATG-3′
	Reverse: 5′-GTCACAGGTGAGGTTCACTGTTTC-3′
	Probe: 5′FAM-CTGGACTCCCGATGCCCCTGG-BHQ-1 3′
IFN-γ	Forward: 5′-GCTTTGCAGCTCTTCCTCATG-3′
	Reverse: 5′-CTTCCACATCTATGCCACTTGAG-3′
	Probe: 5′FAM-CTGTTTCTGGCTGTTACTGCCACGGC-BHQ-1 3′
GATA-3	Forward: 5′-GGTCAAGGCAACCACGTC-3′
	Reverse: 5′-CATCCAGCCAGGGCAGAG-3′
	Probe: 5′FAM-CGCCCGCCTCTGCTGCACG-BHQ-1 3′
T-bet	Forward: 5′-ATTGCCCGCGGGGTTG-3′
	Reverse: 5′-GACAGGAATGGGAACATTCGC-3′
	Probe: 5′FAM-CTGGGAAGCTGAGAGTCGCGCTCA-BHQ-1 3′

Amplification was carried out in a total volume of 20 μl containing 2 μl of 10 × PCR buffer, 2 μl of MgCl_2_ (25 mM), 2 μl of dNTPmix (2.5 mM), 0.4 μl of Tag DNA polymerase (Takara, China), 2 μl of cDNA template, 2 μl (5 μM) of each target gene and β-actin specific primers, 1 μl (5 μM) of target gene and β-actin specific probes. Reaction conditions were the standard conditions for the TagMan PCR (15 s denaturation at 95°C, 30 s annealing at 60°C) with 45 PCR cycles. Relative quantification between samples was achieved by the 2^-ΔΔCT^ method [42] and calculated by software REST 2005 (gifted by Eppendorf company), and is reported as the n-fold difference relative to target gene mRNA expression in the calibrator group (the group of mice immunized with saline) [43].

### MicroRNAs expressed by macrophages stimulated in vitro by docetaxel

The murine macrophage-like cell line RAW264.7 (Invitrogen) was cultured in 24-well plastic plates (Greiner) at a concentration of 2 × 10^6^ cells/ml in High Glucose DMEM (Hyclone) supplemented with 10% FCS and in an atmosphere of 90% humidity containing 5% CO_2_ at 37°C. After the adherent cells were formed, docetaxel solution was added in triplicates to produce a final concentration of 0.8 μg/ml. The cells were harvested after 1 and 3 h incubation for RNA isolation. Expression levels of microRNAs (miR-181a, miR-155, miR-150, miR-146a and miR-125b) were detected according to our lab previously described by Yuan et al. [21]. The primers for microRNA gene were described in Table 2.

**Table 2:** Sequences of primer for quantitative RT-PCR of microRNA

**MicroRNAs**	**Primer sequence**
*miR-181a*	Forward: 5′-AACATTCAACGCTGTCGGTG-3′
*miR-155*	Forward: 5′-GGGGTTAATGCTAATTGTGATAGG-3′
*miR-150*	Forward: 5′-TCTCCCAACCCTTGTACCA-3′
*miR-146a*	Forward: 5′-GGGTGAGAACTGAATTCCATGG-3′
*miR-125b*	Forward: 5′-TCCCTGAGACCCTAACTTGTGA-3′
*Universal reverse primer*	Reverse primer: 5′-GCGAGCACAGAATTAATACGACTC-3′
*Reverse transcription primer*	Poly (T) adapter: 5′-GCGAGCACAGAATTAATACGACTCACTATAGG (T)12VN-3′ (V = A,G,C; N = A,T,G,C) [Virginie Olive 2009]

### Statistical analyses

Data are expressed as mean ± standard deviations (S.D.) in addition to HI titers which are expressed as geometrical mean titer (GMT) ± standard deviations (S.D.) Boniferroni method was used to compare the parameters between groups by SPSS16.0. *P*-values of less than 0.05 were considered statistically significant.

## Abbreviations

Ig, Immunoglobulin; HA, Hemagglutinin; HI, Hemagglutination inhibition; IFN-gamma (IFN-γ), Interferon-gamma; IL, Interleukin; FCS, Foetal calf serum; Con A, Concanavalin A; LPS, Lipopolysaccharide; DMSO, Dimethyl sulfoxide; ELISA, Enzyme linked immunosorbant assay; SI, Stimulation index.

## Competing interests

The authors declare that they have no competing interests.

## Authors’ contributions

JC and LY conceive the method study. SH was responsible for the study design. JC, QF, FS and YC executed the experiments and JC drafted the manuscript. JC and SH analyzed the data and critically revised the manuscript. All authors read and approved the final manuscript. All authors read and approved the final manuscript.
